# Strigolactones Negatively Regulate Tobacco Mosaic Virus Resistance in *Nicotiana benthamiana*

**DOI:** 10.3390/ijms25158518

**Published:** 2024-08-04

**Authors:** Renyan Huang, Shuaijun Bie, Shan Li, Bin Yuan, Li Zhang, Zhuo Zhang, Jianbin Chen, Weimin Ning, Jing Peng, Yu Zhang, Songbai Zhang, Yong Liu, Deyong Zhang

**Affiliations:** 1 Hunan Plant Protection Institute, Hunan Academy of Agricultural Science, Changsha 410125, China; huangry0106@163.com (R.H.); chenjianbin89@126.com (J.C.);; 2 College of Plant Protection, Hunan Agricultural University, Changsha 410128, China; bsj010208@outlook.com; 3 Insititute of Plant Protection and Soil Fertilizer, Hubei Academy of Agricultural Sciences, Wuhan 430064, China; 4 Nuclear Agriculture and Space Breeding Research Institute, Hunan Academy of Agricultural Sciences, Changsha 410125, China

**Keywords:** strigolactones, tobacco mosaic virus, RNA sequencing, plant hormones, plant immunity

## Abstract

Strigolactones (SLs) are plant hormones that regulate diverse developmental processes and environmental responses in plants. It has been discovered that SLs play an important role in regulating plant immune resistance to pathogens but there are currently no reports on their role in the interaction between *Nicotiana benthamiana* and the tobacco mosaic virus (TMV). In this study, the exogenous application of SLs weakened the resistance of *N. benthamiana* to TMV, promoting TMV infection, whereas the exogenous application of Tis108, a SL inhibitor, resulted in the opposite effect. Virus-induced gene silencing (VIGS) inhibition of two key SL synthesis enzyme genes, *NtCCD7* and *NtCCD8*, enhanced the resistance of *N. benthamiana* to TMV. Additionally, we conducted a screening of *N. benthamiana* related to TMV infection. TMV-infected plants treated with SLs were compared to the control by using RNA-seq. The KEGG enrichment analysis and weighted gene co-expression network analysis (WGCNA) of differentially expressed genes (DEGs) suggested that plant hormone signaling transduction may play a significant role in the SL–TMV–*N. benthamiana* interactions. This study reveals new functions of SLs in regulating plant immunity and provides a reference for controlling TMV diseases in production.

## 1. Introduction

Strigolactones (SLs) are a class of carotenoid-derived terpenoid lactones initially characterized as stimulating substances that promote germination of root parasitic plants, such as witchweeds (*Striga* spp.) [1]. The core members of the synthetic pathways of SLs are *D27*, *CCD7*, *CCD8*, and *CYP711A*, among which *CCD7* and *CCD8* are the most important and have been studied the most in various species. The *d17* and *d10* mutants in rice and *max2* and *max3* in Arabidopsis, lacking *CCD7* and *CCD8*, respectively, exhibit a typical SL-deficient phenotype [2,3]. *D27*, both in Arabidopsis and rice, is a SL synthesis enzyme that catalyzes a reversible reaction discovered through an excellent biochemical analysis in vitro [4,5]. Arabidopsis *max1*, lacking in *CYP711A1*, exhibited a hyper-branching phenotype similar to that of *max3* and *max4* [6].

Strigolactones (SLs) play many important regulatory roles in plants, including controlling lateral bud outgrowth [7], regulating the interaction between roots and soil microorganisms [8], and possibly participating in signal transmission between plants, thus affecting their development and reproduction [9,10]. They may also have potential applications in controlling plant pests and diseases and improving plant growth rates. For example, knockout of Arabidopsis *MORE AXILLARY GROWTH2* (*MAX2*), a key component of SLs, reduced resistance to *Pseudomonas syringae* and increased susceptibility to hemi-biotroph pseudomonas syringae and bacterial necrotrophy *Botrytis cinerea* [11]. The silencing of SL biosynthetic genes *CCD7* and *CCD8* in tomatoes enhanced the response to root-knot nematodes [12] and the key biosynthesis genes *GbCCD7* and *GbCCD8b* positively regulate resistance to Verticillium wilt in upland cotton (*Gossypium hirsutum*) [13]. However, whether SLs regulate the resistance of *N. benthamiana* to TMV remains unknown.

The main hormones present in plants include auxins (IAAs), salicylic acid (SA), jasmonic acid (JA), gibberellins (GAs), ethylene (ET), abscisic acid (ABA), cytokinins (CKs), brassinosteroids (BRs) and strigolactones (SLs). These hormones play important roles in plant response to biotic and abiotic stressors and recent studies have provided substantial evidence for their crosstalk. For example, SA has been found to play a major role in the activation of the immune response to biotrophic and hemi-biotrophic pathogens [14] while ET and JA are responsible for resistance to necrotrophic pathogens and herbivorous insects [15,16]. Moderate upregulation of auxin signaling inhibits the burst of reactive oxygen species (ROS) in the early defense response triggered by the PRAM (plant immune-inducing molecule) [17]. The guanine-induced resistance of rice and Arabidopsis to pathogens depends on ethylene and jasmonic acid signaling pathways and is independent of the SA signaling pathway [18]. The BR signal interacts with other immune-related signals to construct a complex signaling network that regulates the interaction between plants and microbes and their ability to adapt to adverse environments [19]. These hormones have been reported to regulate plant response to viral pathogens, such as ABA and ET hormones, both inhibiting TMV infection, and auxin and BRs, both promoting RBSDV infection [20]. The tobacco mosaic virus (TMV) is a single-stranded RNA virus that affects a wide range of plants, such as tobacco and various solanaceous and cruciferous species [21,22,23,24,25]. When a plant contracts TMV, the virus persists indefinitely, causing symptoms like leaf deformation, curling, and mottling, as well as reduced plant size and stunted growth, and this results in decreased yields and economic losses [26,27]. There is currently no report on the involvement of SLs in regulating plant resistance to viral pathogens.

Transcriptome sequencing (RNA-seq) has strongly accelerated research on hormones that influence plant responses to various biotic and abiotic stresses. For example, transcriptome analysis has revealed that the exogenous application of SA can improve the freezing stress tolerance of alfalfa [28]. Transcriptome analysis has revealed the crucial role of the exogenous application of BL in the biosynthesis of alkaloids in *Pinellia ternate* [29]. Integrated transcriptome analysis reveals that plant hormones, such as jasmonic acid and salicylic acid, play an important role in coordinating growth and defense responses following fungal infection in poplar [30]. However, no transcriptome studies have examined SL-mediated virus resistance in *N. benthamiana*.

In this study, we thoroughly investigated the role of SLs in the *N. benthamiana* resistance to TMV by using multiple approaches. First, we showed that the exogenous application of SLs dampened the resistance of tobacco to TMV and the exogenous application of SL biosynthesis inhibitor Tis108 enhanced the resistance of *N. benthamiana* to TMV. Second, the suppression of SL biosynthesis by knocking down the SL genes *NtCCD7* and *NtCCD8* also increased the resistance of *N. benthamiana* to TMV. Furthermore, transcriptome analysis revealed that genes involved in SL synthesis and regulation pathways and another hormone pathway were differentially expressed during the process of affecting resistance to TMV after spraying with SLs. Together, based on these findings, it can be concluded that SLs may negatively regulate the *N. benthamiana* defense against TMV through crosstalk with other hormone signaling pathways. Therefore, studying the effects of SLs on TMV infection promises to provide new avenues for the prevention and control of TMV damage in production.

## 2. Results

### 2.1. SLs Facilitating TMV Infection in N. benthamiana

**Figure 1:**
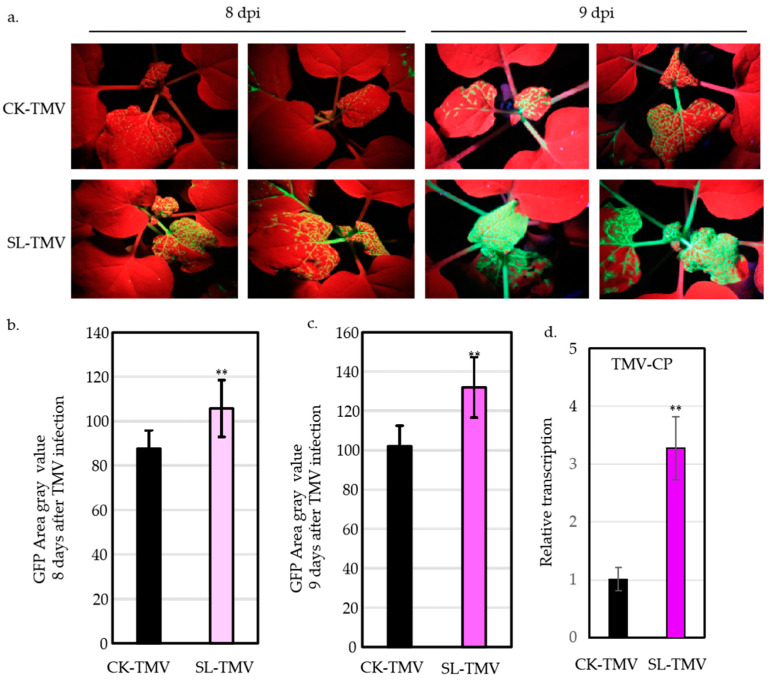
SLs promoting TMV infection in *N. benthamiana*. (**a**) Analysis of GFP fluorescence in TMV-GFP-inoculated leaves after SL application. Images were obtained under a UV light at 8 and 9 days post-inoculation (dpi). (**b**) Statistical quantification of the infection areas on the inoculated leaves at 8 dpi. (**c**) Statistical quantification of the infection areas on the inoculated leaves at 9 dpi. (**d**) Statistical quantification of the experiment of TMV-CP on the inoculated leaves at 9 dpi. Values represent the mean and ± SE of at least *n* = 9 biological replicates from two independent experiments in (**b**,**c**) and *n* = 3 biological replicates from two independent experiments in (**d**); double asterisk in (**b**–**d**) indicate significant differences, as determined by a two-way ANOVA text (*p* < 0.01).CK-TMV, inoculated TMV; SL-TMV, inoculated TMV spraying with SL.

Glasshouse experiments were conducted in order to determine the effect of SLs on TMV of *Nicotiana benthamiana*. One day after spraying with SLs, the plants were inoculated with TMV-GFP again. Plants sprayed with ddH_2_O before TMV-GFP inoculation served as controls. At 8 and 9 days post-TMV-GFP inoculation (dpi), the plants sprayed with SLs displayed much more fluorescence compared with the control plants (Figure 1a). The grayscale area of GFP was obtained by converting all fluorescent photos to area ratios. The results showed that the mean grayscale areas reached 105.67 and 131.93 on the 8th and 9th days after spraying with SL hormones while the control had mean grayscale areas of only 87.53 and 101.97 (Figure 1b,c). We quantified the conserved regions of the TMV virus using the qPCR method and found that the content of the TMV significantly increased after spraying with SL (Figure 1d).

### 2.2. SL Inhibitor Tis108 Suppresses TMV Infection in N. benthamiana

**Figure 2:**
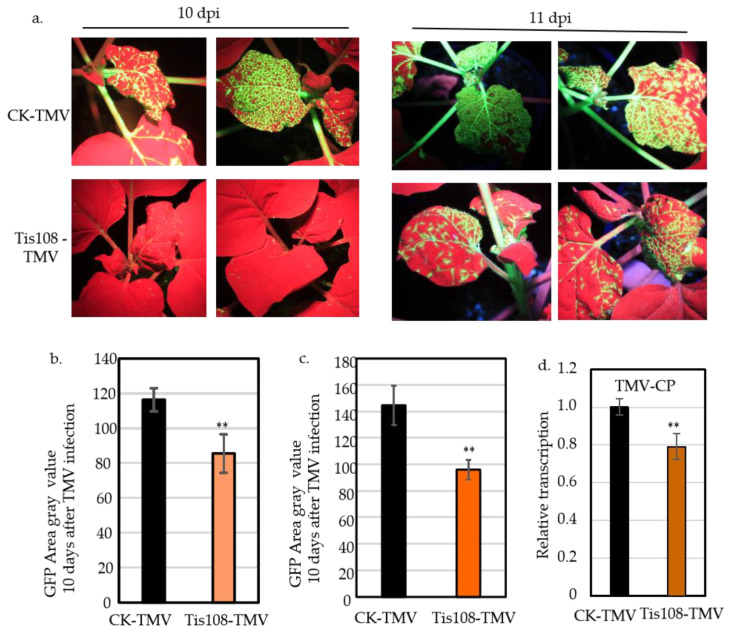
SL inhibitor Tis108 suppresses TMV infection in *N. benthamiana*. (**a**) Analysis of GFP fluorescence in TMV-GFP-inoculated leaves after Tis108 application. Images were obtained under a UV light at 10 and 11 days post-inoculation (dpi). (**b**) Statistical quantification of the infection areas on the inoculated leaves at 10 dpi. (**c**) Statistical quantification of the infection areas on the inoculated leaves at 11 dpi. (**d**) Statistical quantification of the experiment of TMV-CP on the inoculated leaves at 9 dpi Values represent the mean and ± SE of at least *n* = 17 biological replicates from two independent experiments in (**b**,**c**) and *n* = 3 biological replicates from two independent experiments in (**d**); double asterisk in (**b**–**d**) indicate significant differences, as determined by a two-way anova text (*p* < 0.01). CK-TMV, inoculated TMV; SL-TMV, inoculated TMV spraying with SL.

To further validate the impact of SLs on TMV infection, the specific SL inhibitor Tis108 was included in our experimental system. One day after spraying with Tis108, the plants were inoculated with TMV-GFP again. Plants sprayed with ddH_2_O before TMV-GFP inoculation served as controls. At 10 and 11 days post-TMV-GFP inoculation (dpi), the plants treated with Tis108 displayed a smaller fluorescent area compared with the control plants (Figure 2a). The grayscale area of GFP was obtained by converting all fluorescent photos to area ratios. The results showed that the mean grayscale areas were only 85.44 and 96.04 on the 10th and 11th days after spraying with the SL inhibitor Tis108 while the control mean grayscale areas reached 116.31 and 144.80 (Figure 2b,c). Using the qPCR quantitative method to detect the conserved regions of the TMV, we found that the content of the TMV significantly decreased after spraying with the SL inhibitor Tis108 (Figure 2d).

### 2.3. Suppression of NtCCD7 and NtCCD8 Expression in N. benthamiana Plants Dampens TMV Infection

**Figure 3:**
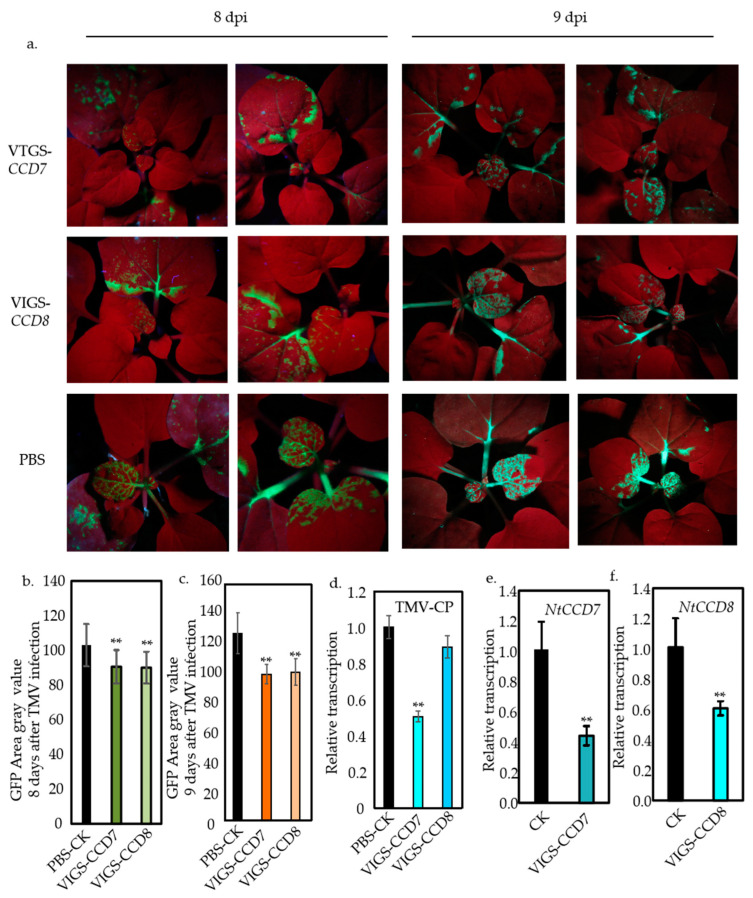
Suppression of *NtCCD7* and *NtCCD8* expression in *N. benthamiana* through VIGS affects TMV infection. (**a**) Analysis of GFP fluorescence in TMV-GFP-inoculated leaves between VIGS-*NtCCD7*, VIGS-*NtCCD8*, and control plants. Images were obtained under a UV light at 8 and 9 days post-inoculation (dpi). (**b**) Statistical quantification of the infection areas on the inoculated leaves at 8 dpi. (**c**) Statistical quantification of the infection areas on the inoculated leaves at 9 dpi. (**d**) Ralation expression of TMV-CP in VIGS-*NtCCD7* and VIGS-*NtCCD8* plants. (**e**) Relative expression of *NtCCD7* in VIGS-*NtCCD7* plants. (**f**) Relation expression of *NtCCD8* in VIGS-*NtCCD8* plants. Values represent the mean and ±SE of at least *n* = 16 biological replicates from two independent experiments in (**b**,**c**). Values represent the mean and ±SE of at least *n* = 3 biological replicates from two independent experiments in (**d**–**f**); double asterisk in (**b**–**f**) indicate significant differences, as determined by a two-way ANOVA text (*p* < 0.01). PBS, control plant; VIGS-*CCD7*, the plant suppressed of *NtCCD7*; VIGS-*CCD8*, the plant suppressed of *NtCCD8*.

In order to further verify the influence of SL hormones on TMV infection in *N. benthamiana*, we knocked down the two key genes, *NtCCD7* and *NtCCD8*, for SL synthesis using VIGS. The expression of *NtCCD7* and *NtCCD8* in the TRV-*NtCCD7*- and TRV-*NtCCD8*-inoculated plants was significantly reduced (Figure 3e,f). We conducted a TMV infection experiment two days after TRV infection and found that plants with inhibited *NtCCD7* and *NtCCD8* expression had significantly lower TMV infection levels on the 8th and 9th days after TMV infection compared with control plants (Figure 3a). The grayscale area of GFP was obtained by converting all fluorescent photos to area ratios. The results showed that the mean grayscale areas of TRV-*NtCCD7* were only 90.63 and 99.09 on the 8th and 9th days and the mean grayscale areas of TRV-*NtCCD8* were only 90.04 and 100.26 on the 8th and 9th days while the control plant had mean grayscale areas that reached 103.08 and 126.73 (Figure 3b,c). Similarly, we used the qPCR quantitative method to detect the conserved regions of the TMV virus and found that inhibiting the two key synthetic genes for SL, *NtCCD7*, and *NtCCD8*, resulted in a decrease in TMV content (Figure 3d).

### 2.4. Identification of the Influence of DEGs of SLs on TMV Infection

**Figure 4:**
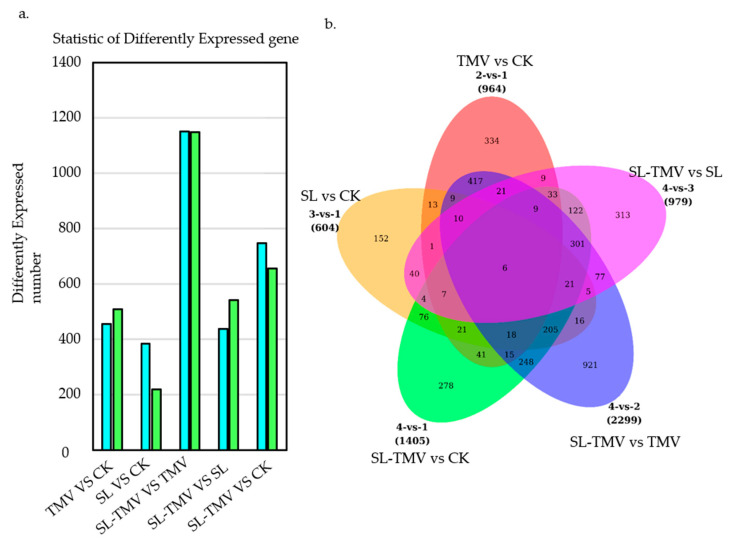
Statistical analysis of differentially expressed unigenes (DEGs) in different comparison groups in *Nicotiana benthamiana*. (**a**) Statistical analysis of up/downregulated unigenes in CK, SL, TMV, and SL-TMV groups. (**b**) Venn diagram of all DEGs. CK, healthy control without TMV inoculation or SL pretreatment; SL, healthy control with SL pretreatment; TMV, TMV inoculation without SL pretreatment; SL-TMV, TMV inoculation with SL pretreatment. CK, control plants; SL, the plants spaied with SL; TMV, the plants inoculated with TMV; SL-TMV, the plants inoculated with TMV after spaying with SL.

RNA-seq analysis was carried out to explore the DEGs using four different treatments (CK, SL, TMV, SL-TMV) to investigate the possible regulatory mechanism of SLs in the *N. benthamiana* response to TMV infection. A total of 561.54 million raw reads were generated from 12 samples, ranging from 42.54 to 50.93 million for each sample. All raw transcriptome sequences were deposited in the National Center for Biotechnology Information (NCBI) sequence read archive (accession number: GSE272661). A total of 42.04 to 50.26 million clean reads were obtained after filtering, with GC content between 43.71% and 44.11% and Q30 ≥ 95.2% (Table S2). There were 3743 DEGs analyzed in five comparison groups (SL vs. CK, TMV vs. CK, SL-TMV vs. SL, SL-TMV vs. TMV, and SL-TMV vs. CK) (Table S3). SL vs. CK had the fewest DEGs, with 384 upregulated genes and 220 downregulated genes. The SL-TMV vs. TMV group had the highest number of DEGs, including 1152 upregulated genes and 1147 downregulated genes. The TMV vs. CK, SL-TMV vs. SL, and SL-TMV vs. CK groups also had relatively large numbers of DEGs: 964, 979, and 1405, respectively (Figure 4a and Table S4). Only six DEGs were shared across all five groups and there were more DEGs in the TMV vs. CK and SL-TMV vs. TMV groups than in the other three groups (Figure 4b).

### 2.5. GO Functional Annotation and KEGG Pathway Analysis of SLs’ Influence on TMV Infection

**Figure 5:**
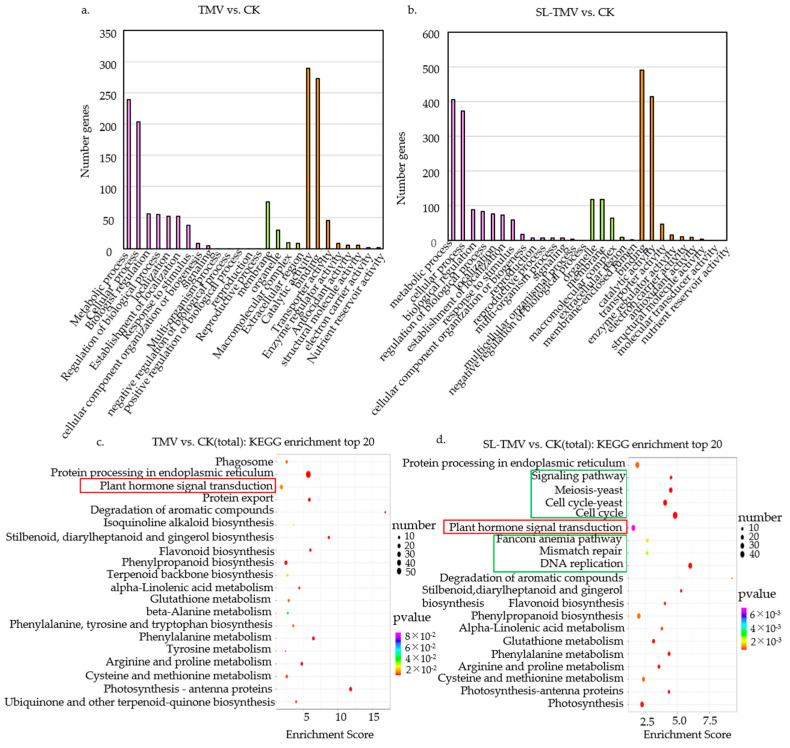
GO and KEGG analysis of DEGs in different comparison groups in *Nicotiana benthamiana*. (**a**) TMV vs. CK, (**b**) SL-TMV vs. CK, (**c**) TMV vs. CK, and (**d**) SL-TMV vs. CK. Each figure shows the top 20 pathways with Q values. CK, healthy control without TMV inoculation or SL pretreatment; SL, healthy control with SL pretreatment; TMV, TMV inoculation without SL pretreatment; SL-TMV, TMV inoculation with SL pretreatment. CK, control plants; SL, the plants spaied with SL; TMV, the plants inoculated with TMV; SL-TMV, the plants inoculated with TMV after spaying with SL.

The GO enrichment analysis of the 964 DEGs in the TMV vs. CK group showed that the most abundant terms in the categories of biological processes were metabolic process, cellular process, and biologist regulation. The other most enriched categories were the membrane, organelle, and macromolecular complex in the category of cell components and catalytic activity and binding in the category of molecular function (Figure 5a). Compared with the TMV vs. CK group, the SL vs. CK group and the SL-TMV vs. CK group had unique GO terms, including membrane-enclosed lumen and molecular transducer activity, and the SL-TMV vs. CK group had unique GO terms of the multicellular organismal process not in the TMV vs. CK group and the SL vs. CK group (Figure 5b and Supplementary Figure S1a).

KEGG analysis of the TMV vs. CK and SL-TMV vs. CK groups showed that plant hormone signal transduction, flavonoid biosynthesis, and phenylalanine metabolism were enriched (Figure 5c). This indicates that these co-enriched pathways may play important roles in the process of TMV infection. Interestingly, signaling pathways, meiosis, the cell cycle, the Fanconi anemia pathway, mismatch repair, and DNA replication were specifically enriched in the SL-TMV vs. CK group (indicated by the green box) (Figure 5d). It is noteworthy that both are enriched in the plant hormone signaling pathway except the SL-TMV vs. SL group (indicated by the red box) (Figure 5c,d and Supplementary Figure S1b,d,f). This suggested that these pathways may play a role in modifying the extent of TMV infection after spraying with SLs.

### 2.6. Venn Diagram Analysis of DEGs in the TMV vs. CK and SL-TMV vs. CK Groups

**Figure 6:**
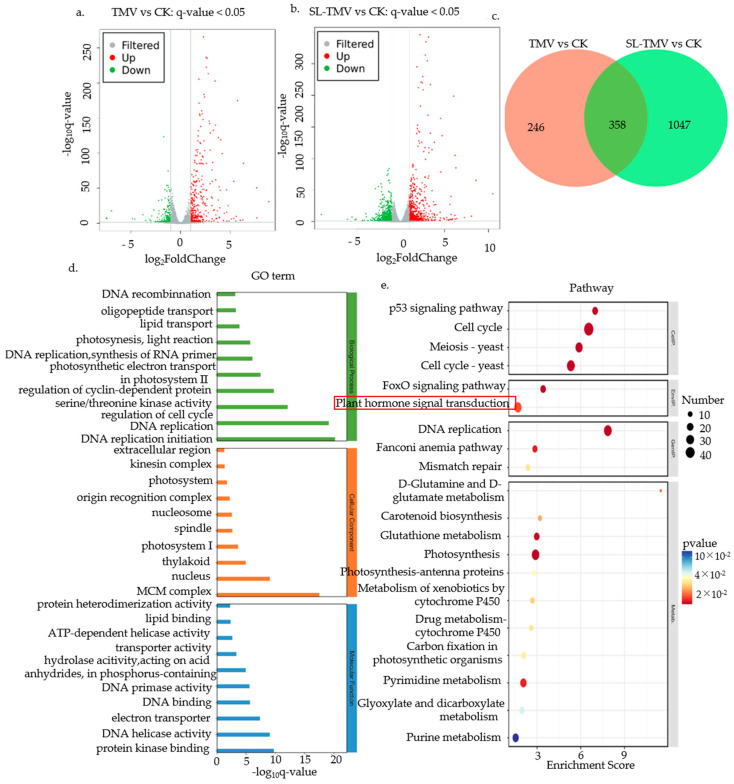
Comparative analysis of differences between the TMV vs. CK and SL-TMV vs. CK groups. (**a**) The differentially expressed genes in the TMV vs. CK group. (**b**) The differentially expressed genes in the SL-TMV vs. CK group. (**c**) Venn diagram of DEGs in the TMV vs. CK and SL-TMV vs. CK groups. (**d**) GO analysis of 1047 DEGs. (**e**) KEGG analysis of 1047 DEGs. CK, control plants; TMV, the plants inoculated with TMV; SL-TMV, the plants inoculated with TMV after spaying with SL.

Up-regulated genes were more prevalent than downregulated genes in the TMV vs. CK and SL-TMV vs. CK groups (Figure 6a,b). A total of 358 DEGs were shared between the TMV vs. CK and SLTMV vs. CK groups (Figure 6c). These DEGs might be closely related to the response to the TMV infection. Interestingly, a total of 1047 DEGs were unique in the SLTMV vs. CK group but not in the TMV vs. CK group as a result of the application of SLs (Figure 6c). In the GO analysis, they were enriched in protein kinase binding, DNA helicase activity, and electron transporters (Figure 6d). In the KEGG pathway analysis, these DEGs were enriched in plant hormone signal transduction, carotenoid biosynthesis, and photosynthesis (Figure 6e).

### 2.7. Gene Co-Expression Network Analysis of SLs’ Influence on TMV Infection

**Figure 7:**
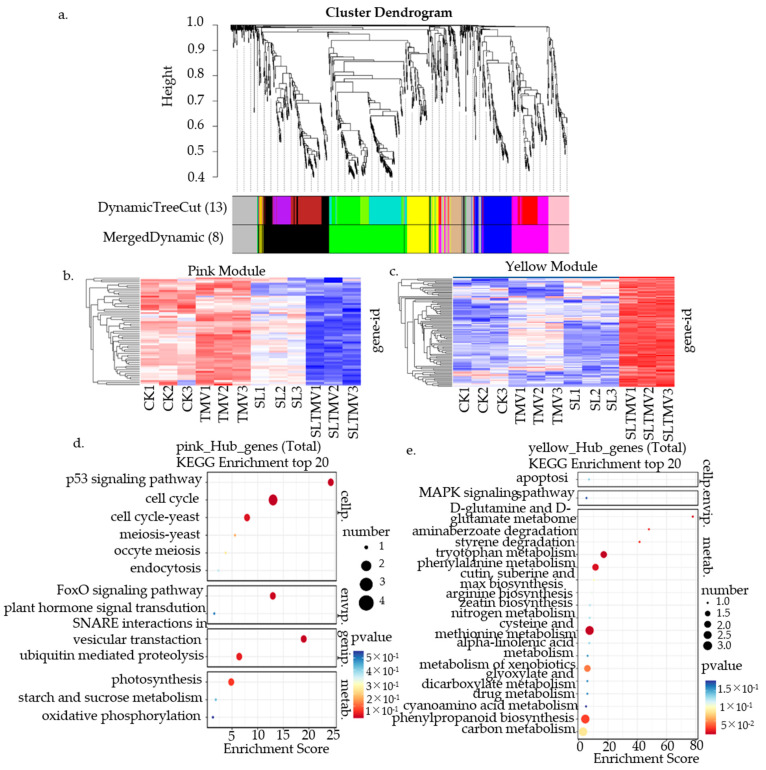
Weighted gene co-expression network analysis. (**a**) Gene cluster dendrograms and module division. (**b**) The expression heatmap and eigenvalues of genes in the pink module. (**c**) The expression heatmap and eigenvalues of genes in the yellow module. (**d**) The correlation networks of hub genes corresponding to the pink modules. (**e**) The correlation networks of hub genes corresponding to the yellow modules. CK, control plants; SL, the plants spaied with SL; TMV, the plants inoculated with TMV; SL-TMV, the plants inoculated with TMV after spaying with SL.

Weighted gene co-expression network analysis (WGCNA) is a systems biology approach used to identify co-expressed gene networks within a large set of genes. Eight different modules were obtained using a gene dendrogram colored according to correlations between gene expression levels (Figure 7a). The genes in the pink module and yellow module were inversely expressed in the TMV and SL-TMV. We performed KEGG analysis for these two modules. In the pink module, pathways related to the “p53 signaling pathway”, “cell cycle”, and “plant hormone signal transduction” were enriched. In the yellow module, pathways related to “cysteine and methionine metabolism”, “phenylpropanoid biosynthesis”, and the “MAPK signaling pathway” were enriched (Figure 7b–e). Therefore, these disease-resistance genes and plant hormone signal transduction genes should be studied in depth to elucidate their role in the SL-mediated susceptibility response to TMV infection in *N. benthamiana*.

### 2.8. DEGs Related to Plant Hormone Signal Transduction 

**Figure 8:**
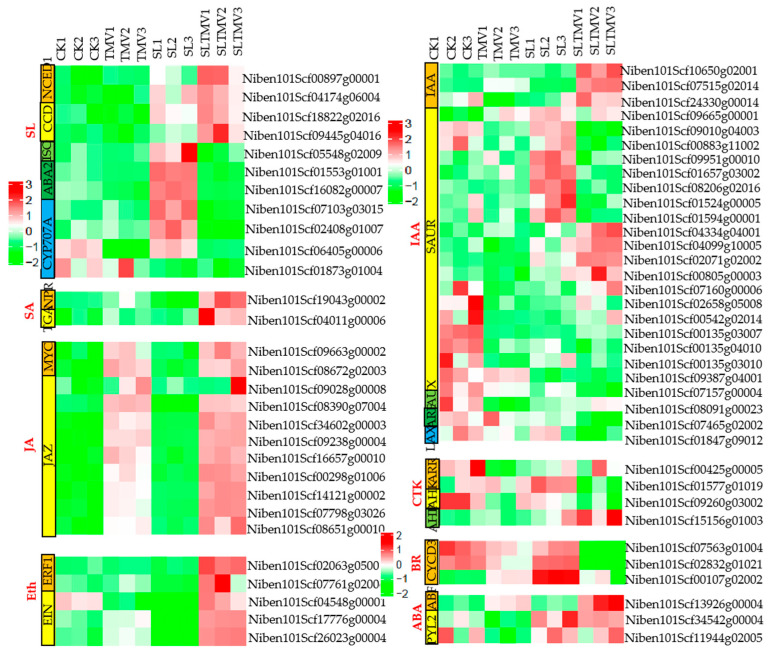
Expression of plant hormone signal transduction. CK, control plants; SL, the plants spaied with SL; TMV, the plants inoculated with TMV; SL-TMV, the plants inoculated with TMV after spaying with SL.

DEGs in hormone signaling pathways were screened out to study the impact of spraying with SLs on the influence of *N. benthamiana* on TMV. Among these DEGs, the largest number belongs to the IAA signaling pathway, with a total of 26 genes, mainly encoding the SAUR family protein. Secondly, the SL and JA signaling pathways are both represented by 11 DEGs, with the SL pathway primarily characterized by genes encoding CYP707A, NCED1, CCD, and ABA2, while the JA pathway is represented by two families of proteins, JAZ and MYC. Hormones, such as ETH, CTK, BR, ABA, and SA, also contain a small number of differentially expressed genes (Figure 8). This indicates that plant hormones, especially IAA, SL, and JA, play an important role in affecting the infection of TMV in *N. benthamiana* sprayed with SLs.

### 2.9. qRT-PCR Validation of Strigolactone-Signaling-Related Genes

**Figure 9:**
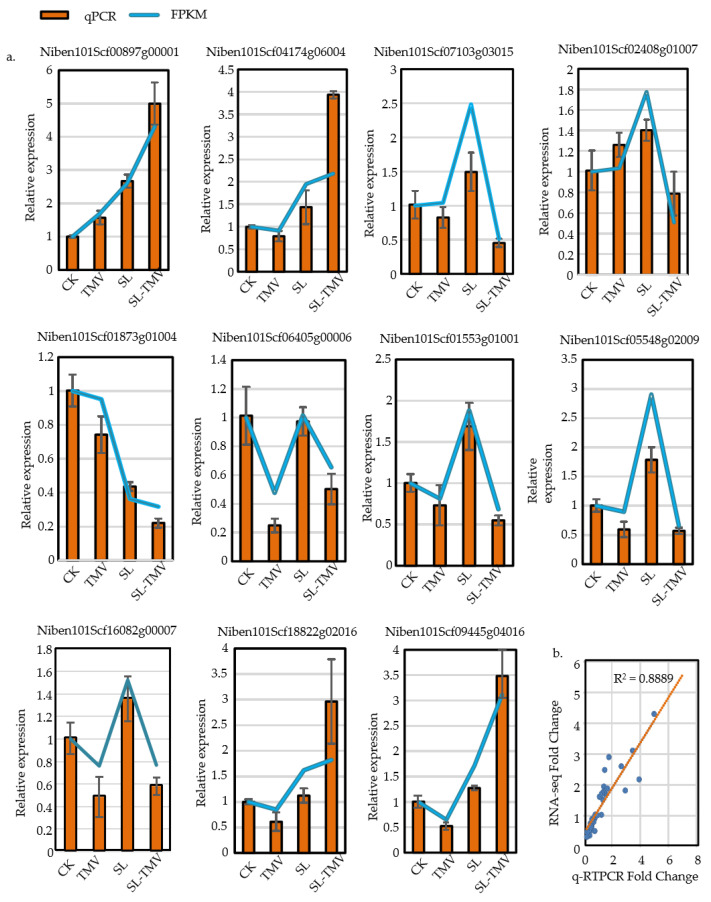
Consistency results of qRT-PCR and RNA-seq. (**a**) Expression of mRNAs by qRT-PCR and RNA-seq. (**b**) Consistency results of qRT-PCR and RNA-seq. The figure exhibits the fold change in the qRT-PCR and RNA-seq results. R2 represents the correlation coefficient between qRT-PCR and RNA-seq, orange line represent correlation coefficient, blue dots represent the ratios of RNA-seq TO qRT-PCR for different genes. CK, control plants; SL, the plants spaied with SL; TMV, the plants inoculated with TMV; SL-TMV, the plants inoculated with TMV after spaying with SL.

To further verify the accuracy and reliability of the RNA-seq results, expression levels of 11 genes in the strigolactone signal transduction pathway following CK, TMV, SL, and SL-TMV treatments were detected via qRT-PCR. In general, the expression data obtained from the qRT-PCR were consistent with the RNA-seq results, indicating a similar trend between qRT-PCR and the transcriptome datasets (Figure 9a). The correlation between the fold change in the qPCR and RNA-seq (FPKM) results for the 11 DEGs was calculated using Pearson correlation and linear regression analyses showed a significantly positive correlation (Figure 9b).

## 3. Discussion

Salicylic acid (SA) was first discovered in 1979 as an elicitor that induces resistance in tobacco plants to tobacco mosaic virus (TMV) [31]. However, exogenously applied methyl jasmonate has been shown to reduce local resistance to TMV and allow systemic viral movement [32]. Both abscisic acid (ABA) and ethylene (ET) negatively regulate TMV infection in tobacco [20]. Exogenous BRs enhance the resistance of plants to virus infection while spraying plants with BR inhibitors increases their susceptibility to viruses [33]. Strigolactones (SLs) serve several crucial regulatory functions in plants, such as managing the outgrowth of lateral buds, influencing the relationship between roots and soil microorganisms, and potentially engaging in interplant signal transmission, thereby impacting plant development and reproduction [6,7,8]. However, the detailed functions and mechanisms modulated by SLs in regulating TMV infection remain unclear in tobacco. In the present study, we demonstrated that SLs negatively regulate tobacco resistance to TMV. First, the exogenous application of SLs reduced the resistance of tobacco to TMV (Figure 1). Second, the application of Tis108, a SL biosynthesis inhibitor, enhanced the resistance of tobacco to TMV (Figure 2). Knockdown of *NtCCD7* and *NtCCD8* also enhanced the resistance (Figure 3). Collectively, our data demonstrate clearly, for the first time, that SLs negatively regulate tobacco resistance to TMV, which extends the biological role of SLs in tobacco.

Generally, SLs cooperate with other hormones to regulate plant responses to biotic or abiotic stresses. For example, SLs positively regulate cotton resistance to Verticillium wilt through cross-interaction with other hormones [13]. SLs enhance the resistance of Arabidopsis thaliana by inducing salicylic-acid-mediated disease resistance [34]. Exogenous SLs contribute to drought acclimatization by enhancing stomata sensitivity to ABA [35]. SL and gibberellin crosstalk regulate rice in response to N availability [36]. In this study, by employing transcriptome KEGG analysis, it was found that specific expressed genes in the SL-TMV vs. CK group were enriched in plant hormone signal transduction pathways. In addition, many genes involved in hormone-related pathways showed changes in expression levels. For example, there are 11 DEGs in both the JA and SA signal transduction pathways. The IAA hormone pathway contains the largest number of DEGs, reaching a total of 26 genes (Figure 8). Additionally, we measured the expression levels of the ABF and PYL2 genes in the ABA pathway and the ERF1 and EIN genes in the Eth pathway via qPCR. We observed that after spraying SL and inoculating TMV, the expression levels of these genes significantly increased (Supplementary Figure S2). It can be seen that SLs may regulate tobacco resistance to TMV through cross-interaction with other hormones.

CCD7 and CCD8 are the most important enzymes involved in the biosynthesis of strigolactones and are the most studied in various species. The *d17* and *d10* mutants in rice and *max2* and *max3*, separately lacking *CCD7* and *CCD8*, exhibit a typical SL-deficient phenotype [2,3]. Based on these findings, we selected *NtCCD7* and *NtCCD8* as targets to interfere with the SL levels in tobacco. As expected, the knockdown of *NtCCD7* and *NtCCD8* enhanced the resistance of tobacco to TMV. In future work, we can conduct in-depth research on *NtCCD7* and *NtCCD8* in other plants. The knockout and overexpression of *NtCCD7* and *NtCCD8* will also be studied.

In conclusion, our results demonstrated that SLs negatively regulate TMV resistance in tobacco. Spraying tobacco plants with inhibitors of SLs can significantly enhance their resistance to TMV and this means that the SL inhibitors may be used in production to prevent and control TMV disease. Fully understanding the influence of the exogenous application of SLs on the interaction between TMV and tobacco will be helpful in developing agricultural measures to prevent and control TMV. Despite many recent insights into the tobacco–TMV interactions using several assays, there are still large knowledge gaps regarding their mechanisms, which require further research in the future, such as focusing on field testing on exogenous SLs and crosstalk among plant signaling pathways.

## 4. Materials and Methods

### 4.1. Virus Sources, Inoculation, and SLs/Tis108 Treatment

SL/Tis108 Spraying: A 1 mM/L stock solution of SL powder (Yuanye Bio-Technology Co. Ltd., Shanghai, China) and Tis108 powder (Rowan, Annapolis, MD, USA) was prepared. Then, the stock solution was diluted 1000-fold with sterile ddH_2_O and 0.015% (*v*/*v*) of Tween 20 was added. A measurement of 200 mL was prepared for spraying and stored for later use. A total of 200 uL of the stock solution was taken and 199.8 mL of ddH_2_O and 30 uL of Tween 20 were added. As the control method, 200 ul of methanol was used instead of the stock solution. On the day before TMV inoculation, SLs/Tis108 and the corresponding control solution were evenly sprayed with a spray bottle onto tobacco leaves, which were then incubated in the dark overnight.

TMV-GFP Inoculation: Both viral infectious clones were introduced into Agrobacterium tumefaciens strain EHA105. *N. benthamiana* (accession: Nb-1) plants were grown in a greenhouse with a 16 h light and 8 h dark cycle at 26 °C. The infiltration of the Agrobacterium with infectious clones into *N. benthamiana* leaves was carried out at the 4–6 leaf stage as previously described [37]. At 7–12 days post-TMV-GFP inoculation (dpi), photos were taken and observations were recorded under fluorescent plant lights. A website (https://cnij.imjoy.io/) was used to convert all fluorescent photos taken into quantitative values. The statistical analysis was determined by a two-way ANOVA text.

### 4.2. VIGS Vector Construction and TRV-Mediated VIGS Assay

The TRV vector construction and introduction of Agrobacterium tumefaciens were performed according to previous methods [38]. Partial sequences of *NtCCD7* (302 bp, Niben101Scf00878g02006.1) and *NtCCD8* (355 bp, Niben101Scf01611g07010.1) were acquired from *N. benthamiana* complementary DNA via a transcription polymerase chain reaction (RT-PCR) with corresponding primers (Supplementary Table S1), cloned to pEASY-T5, and sequenced. The *NtCCD7* and *NtCCD8* fragments were inserted into the tobacco rattle virus (TRV2) using *XbaI* and *BamHI* digestion. TRV2-*NtCCD7* and TRV2-*NtCCD8* were transformed into *Agrobacterium tumefaciens* strain GV3101. The Agrobacterium was grown in 1.00 at 600 nm and mixed 1:1 (*v*/*v*) and infiltration into the leaves of *N. benthamiana* plants was carried out as described [39]. Seven days after agroinfiltration, the young upper leaves were inoculated with TMV.

### 4.3. RNA Extraction, cDNA Library Construction, and Sequencing

Total RNA was obtained 24 h after TMV-GFP inoculation for each group for a total of 12 samples and was used for RNA-seq. Total RNA was extracted using the TRIzol reagent (Invitrogen, Carlsbad, CA, USA) according to the manufacturer’s instructions. The RNA purity and quantity were verified using a NanoDrop 2000 spectrophotometer (Thermo Scientific, Waltham, MA, USA). The RNA integrity was evaluated using an Agilent 2100 Bioanalyzer (Agilent Technologies, Santa Clara, CA, USA). A transcriptome library was constructed using the VAHTS Universal V5 RNA-seq Library Preparation Kit according to the manufacturer’s instructions. Transcriptome sequencing and analysis were performed by OE Biotech Co., Ltd. (Shanghai, China).

### 4.4. RNA Sequencing and Analysis of Differentially Expressed Genes

The libraries were sequenced using the Illumina Novaseq 6000 sequencing platform and 150 bp paired-end reads were generated. Raw reads of the fastq format were processed using fastp (V0.20.1)software and the low-quality reads were removed to obtain clean reads for subsequent data analysis. HISAT2 (V2.1.0) software was used for reference genome alignment and calculating gene expression levels (FPKM) and gene read counts (counts) were obtained via HTSeq-count. R (v 3.2.0) was used for PCA analysis and the plotting of genes (counts) to evaluate the biological replication of samples. DEG analysis was performed using DESeq2 software (version 1.12.3), whereby genes meeting a q value < 0.05 and a foldchange >2 or <0.5 threshold were defined as DEGs. R (v 3.2.0) was utilized for hierarchical clustering analysis of DEGs to show gene expression patterns across different groups and samples. R package ggradar was used to create a radar chart for the top 30 genes to show the expression changes of up- and downregulated genes.

Based on the hypergeometric distribution algorithm, DEGs were analyzed using GO, KEGG pathway, Reactome, and WikiPathways enrichment analysis to screen significantly enriched functional items. R (v 3.2.0) was used to create bar charts, chord diagrams, and enrichment analysis circles for significantly enriched functional items.

### 4.5. Gene Co-Expression Network Analysis

Gene co-expression network analysis was carried out using the WGCNA package (https://cloud.oebiotech.cn). Gene dendrograms were created with colors based on the correlations between the TMV infection area and the expression levels of genes. These gene dendrograms were used to build clustering trees and to divide modules.

### 4.6. qRT-PCR Analysis

To confirm the transcriptome data, 11 candidate genes in the SL signaling pathway were selected for qRT-PCR analysis. The first strand of cDNA was synthesized using an all-in-one 5X RT mastermix kit (abm (New York, NY, USA), Cat. No. G592). The qRT-PCR was conducted with a Roche LightCycler 480 II Real-Time PCR System using BlasTag^TM^ 2X qPCR mastermix (abm, Cat. No. G891, G892) and the procedure was performed under the following conditions: 95 °C for 3 min, followed by 40 cycles of 95 °C for 15s, then 60 °C for 1 min. The qRT-PCR primers used for the candidate gene validation are listed in Table S1. The NtActin gene was used as an internal control (CK). Gene expression was evaluated by using the 2^−△△Ct^ method with three independent biological replicates.

## 5. Conclusions

In our study, we used multiple methods to thoroughly examine how strigolactones (SLs) affect *N. benthamiana*’s resistance to tobacco mosaic virus (TMV). Our findings indicated that both the exogenous application of SLs and suppression of SL biosynthesis (via *NtCCD7* and *NtCCD8* gene knockdown) significantly influence TMV resistance. Transcriptome analysis further revealed DEGs in SL synthesis and regulation pathways, suggesting potential crosstalk with other hormone signaling pathways. These insights into the role of SLs in TMV infection may inform new strategies for preventing and controlling TMV damage.

## Data Availability

The original contributions presented in the study are included in the article, further inquiries can be directed to the corresponding author.

## Supplementary Materials

The following supporting information can be downloaded at: https://www.mdpi.com/article/10.3390/ijms25158518/s1.
