# Supplementary material for: Strigolactones Negatively Regulate Tobacco Mosaic Virus Resistance in Nicotiana benthamiana
Source: Int J Mol Sci. 2024 Aug 4;25(15):8518. doi: 10.3390/ijms25158518 (PMC11313310; doi:10.3390/ijms25158518)
Supplement: Supplementary file 1 [file ijms-25-08518-s001.zip › Supplementary Figures.pptx]

## Slide 1
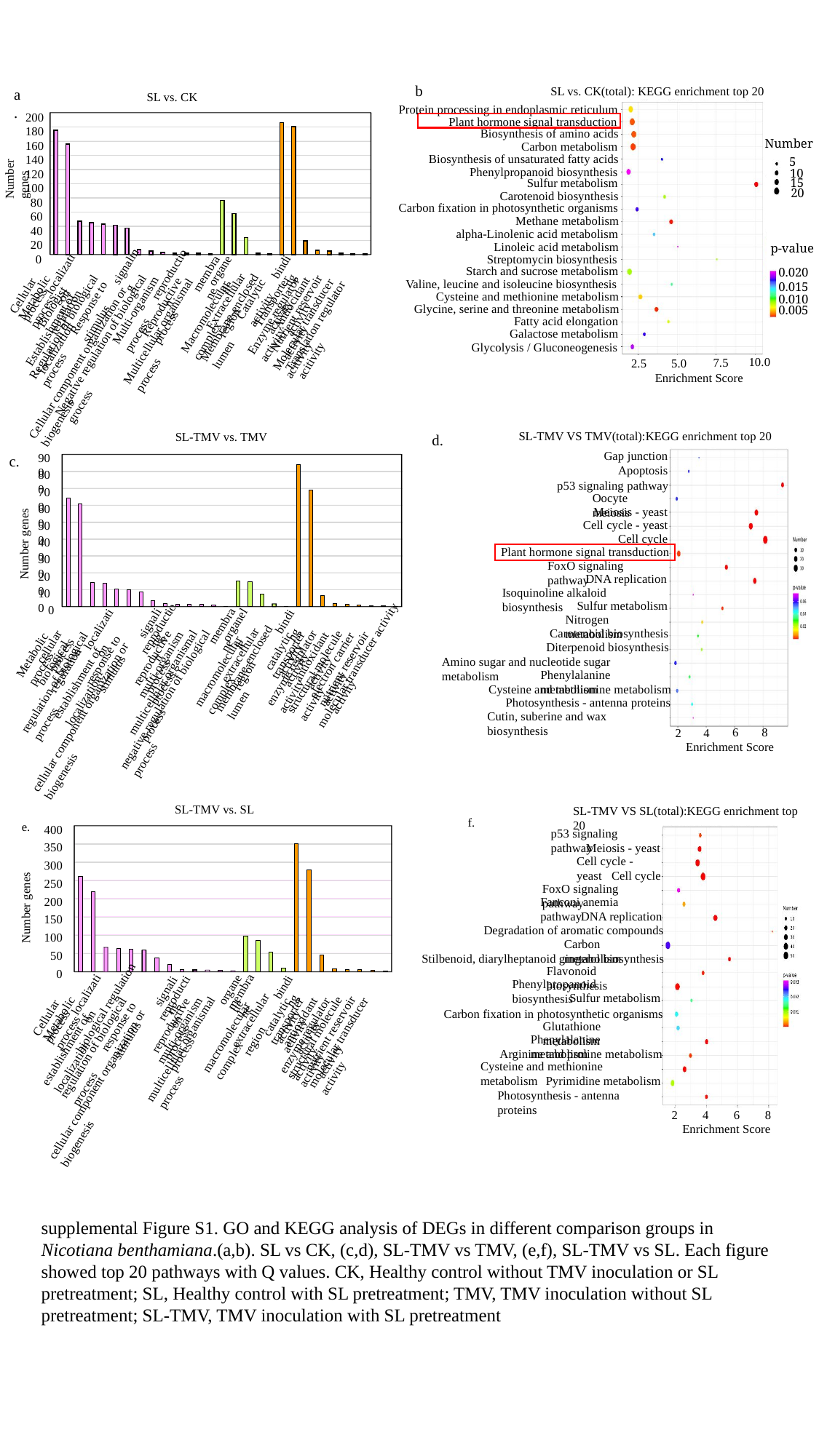

b.
SL vs. CK(total): KEGG enrichment top 20
a.
SL vs. CK
Protein processing in endoplasmic reticulum
Plant hormone signal transduction
200
180
160
140
120
100
80
60
40
20
0
Biosynthesis of amino acids
Number genes
Number
Carbon metabolism
Biosynthesis of unsaturated fatty acids
5
Phenylpropanoid biosynthesis
10
15
Sulfur metabolism
20
Carotenoid biosynthesis
Carbon fixation in photosynthetic organisms
Methane metabolism
Cellular component organization or biogenesis
alpha-Linolenic acid metabolism
Cellular process
Negative regulation of biological grocess
Establishment of localization
Molecular transducer acitivity
Translation regulator acitivity
Reproductive process
Regulation of biological process
Response to stimulus
Transporter activity
Biologist regulation
Enzyme regulator activity
Linoleic acid metabolism
Multi-organism process
Macromolecular complex
Metabolic process
Nutrient reservoir activity
p-value
Antiocidant activity
reproduction
Multicellular organismal process
Extracellular region
Membrane-enclosed lumen
signaling
membrane
Catalytic activity
localization
organelle
binding
Streptomycin biosynthesis
Starch and sucrose metabolism
0.020
Valine, leucine and isoleucine biosynthesis
0.015
Cysteine and methionine metabolism
0.010
Glycine, serine and threonine metabolism
0.005
Fatty acid elongation
Galactose metabolism
Glycolysis / Gluconeogenesis
10.0
7.5
2.5
5.0
Enrichment Score
SL-TMV VS TMV(total):KEGG enrichment top 20
Gap junction
Apoptosis
p53 signaling pathway
Oocyte meiosis
Meiosis - yeast
Cell cycle - yeast
Cell cycle
Plant hormone signal transduction
FoxO signaling pathway
DNA replication
Isoquinoline alkaloid biosynthesis
Sulfur metabolism
Nitrogen metabolism
Carotenoid biosynthesis
Diterpenoid biosynthesis
Amino sugar and nucleotide sugar metabolism
Phenylalanine metabolism
Cysteine and methionine metabolism
Photosynthesis - antenna proteins
Cutin, suberine and wax biosynthesis
6
8
4
2
Enrichment Score
SL-TMV vs. TMV
d.
c.
900
800
700
Number genes
600
500
400
300
200
cellular component organization or biogenesis
structural molecule activity
molecular transducer activity
negative regulation of biological process
100
multi-organism process
antioxidant activity
multicellular organismal process
biological regulation
membrane-enclosed lumen
nutrient reservoir activity
transporter activity
enzyme regulator activity
establishment of localization
reproductive process
reproduction
catalytic activity
response to stimulus
macromolecular complex
extracellular region
electron carrier activity
cellular process
binding
localization
organelle
signaling
membrane
0
regulation of biological process
Metabolic process
SL-TMV vs. SL
SL-TMV VS SL(total):KEGG enrichment top 20
p53 signaling pathway
Meiosis - yeast
Cell cycle - yeast
Cell cycle
FoxO signaling pathway
Fanconi anemia pathway
DNA replication
Degradation of aromatic compounds
Carbon metabolism
Stilbenoid, diarylheptanoid gingerol biosynthesis
Flavonoid biosynthesis
Phenylpropanoid biosynthesis
Sulfur metabolism
Carbon fixation in photosynthetic organisms
Glutathione metabolism
Phenylalanine metabolism
Arginine and proline metabolism
Cysteine and methionine metabolism
Pyrimidine metabolism
Photosynthesis - antenna proteins
8
2
4
6
Enrichment Score
Number genes
f.
e.
400
350
300
250
200
150
100
biological regulation
50
Cellular process
establishment of localization
cellular component organization or biogenesis
structural molecule activity
molecular transducer activity
multicellular organismal process
regulation of biological process
reproductive process
enzyme regulator activity
nutrient reservoir activity
Metabolic process
macromolecular complex
multi-organism process
extracellular region
transporter activity
antioxidant activity
response to stimulus
membrane
catalytic activity
organelle
localization
reproduction
binding
signaling
0
supplemental Figure S1. GO and KEGG analysis of DEGs in different comparison groups in Nicotiana benthamiana.(a,b). SL vs CK, (c,d), SL-TMV vs TMV, (e,f), SL-TMV vs SL. Each figure showed top 20 pathways with Q values. CK, Healthy control without TMV inoculation or SL pretreatment; SL, Healthy control with SL pretreatment; TMV, TMV inoculation without SL pretreatment; SL-TMV, TMV inoculation with SL pretreatment

## Slide 2
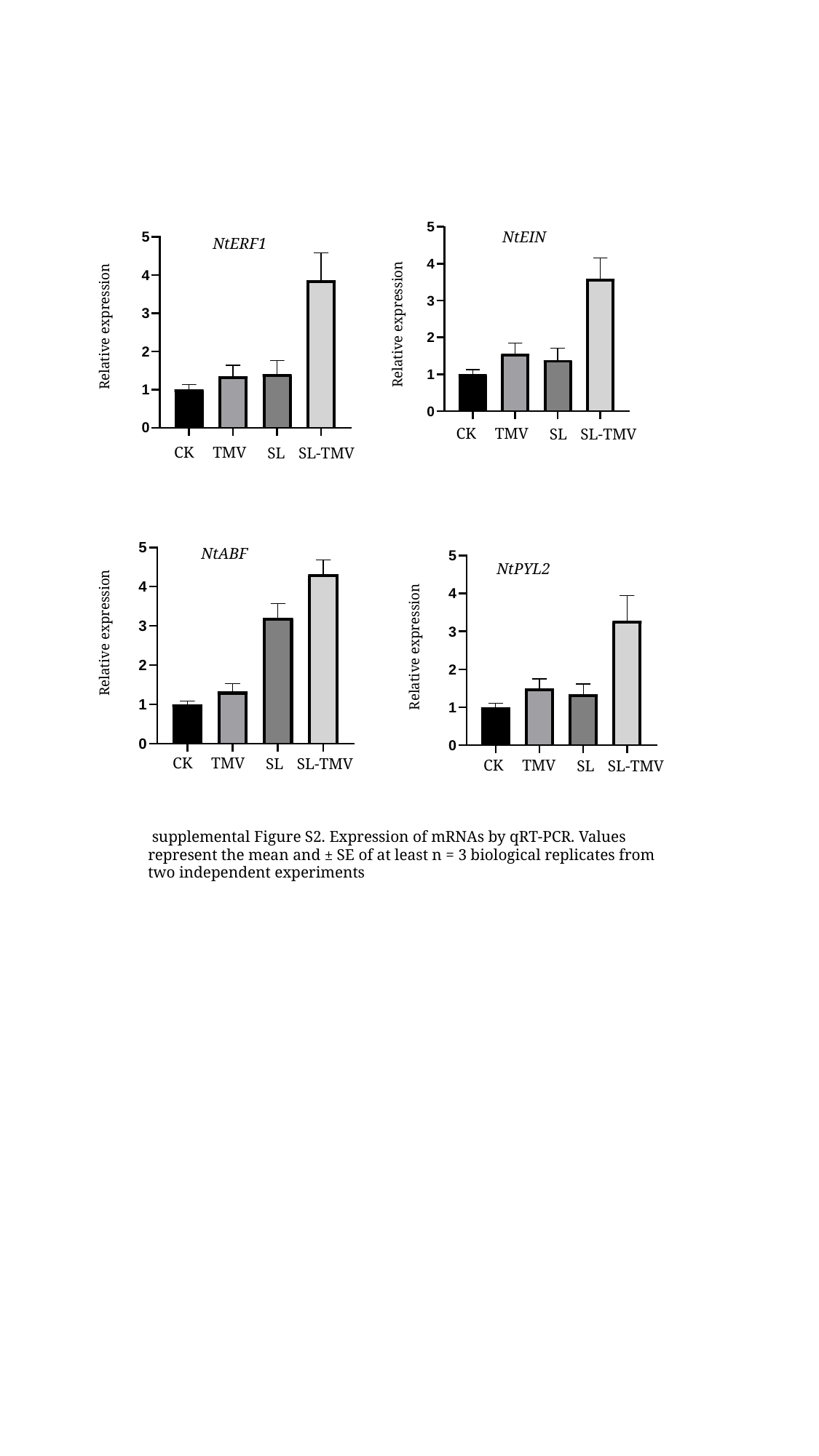

NtEIN
NtERF1
Relative expression
Relative expression
CK
TMV
SL
SL-TMV
CK
TMV
SL
SL-TMV
NtABF
Relative expression
NtPYL2
Relative expression
CK
TMV
SL
SL-TMV
CK
TMV
SL
SL-TMV
 supplemental Figure S2. Expression of mRNAs by qRT-PCR. Values represent the mean and ± SE of at least n = 3 biological replicates from two independent experiments
